# Unusual Extensive Physiologic Pigmentation of the Tongue: A Case Report

**DOI:** 10.7759/cureus.26767

**Published:** 2022-07-12

**Authors:** Manuel Neiva-Sousa, Mariluz Martins, Sandra Bitoque, Delfim Doutel, Pedro Gomes

**Affiliations:** 1 Department of Maxillofacial Surgery, Hospital de São José, Centro Hospitalar Universitário de Lisboa Central, Lisbon, PRT; 2 Department of Head and Neck Surgery, Instituto Português de Oncologia de Lisboa Francisco Gentil, Lisbon, PRT; 3 Department of Pathological Anatomy, Instituto Português de Oncologia de Lisboa Francisco Gentil, Lisbon, PRT

**Keywords:** melanoma, differential diagnosis, melanin, tongue, physiologic pigmentation

## Abstract

The deposition of colored endogenous or exogenous substances in the tissues of the tongue may result in pigmented lesions of the lingual mucosa. The accurate identification of the underlying condition can be difficult to achieve and relies mainly on patient history and clinical and histological evaluation. We present the case of a 30-year-old male referred to our hospital with a chief complaint of extensive pigmentation of the lingual dorsum. A diagnosis of physiologic pigmentation based on clinical and histological findings was made. Since some life-threatening diseases may present solely as pigmented lesions of the tongue, an early diagnosis is of utmost importance.

## Introduction

The accumulation of colored substances in the tissues of the tongue can result in pigmented lesions of the lingual mucosa [1,2]. Pigmentation can be related to either exogenous or endogenous etiologic factors. The former includes drug-induced melanosis, erosion of amalgam, systemic exposure to heavy metals, and tobacco consumption [3,4]. The latter most commonly relates to genetically determined or systemic disease-induced melanin production, as well as hemoglobin, hemosiderin, or bilirubin deposition following submucosal hemorrhage [1,5]. The clinical presentation of lesions may help establish a diagnosis, as multifocal or diffuse macular pigmentation typically relates to physiologic pigmentation or melanosis induced by systemic disease, smoking, or medication, while solitary focal pigmentation more commonly relates to melanotic macule, amalgam tattoo, melanocytic nevus, melanoacanthoma, or melanoma [2]. Regardless, in some cases, this distinction is not so clear. Among differential diagnoses, melanoma is the most life-threatening condition and should always be excluded.

## Case presentation

**Figure 1:**
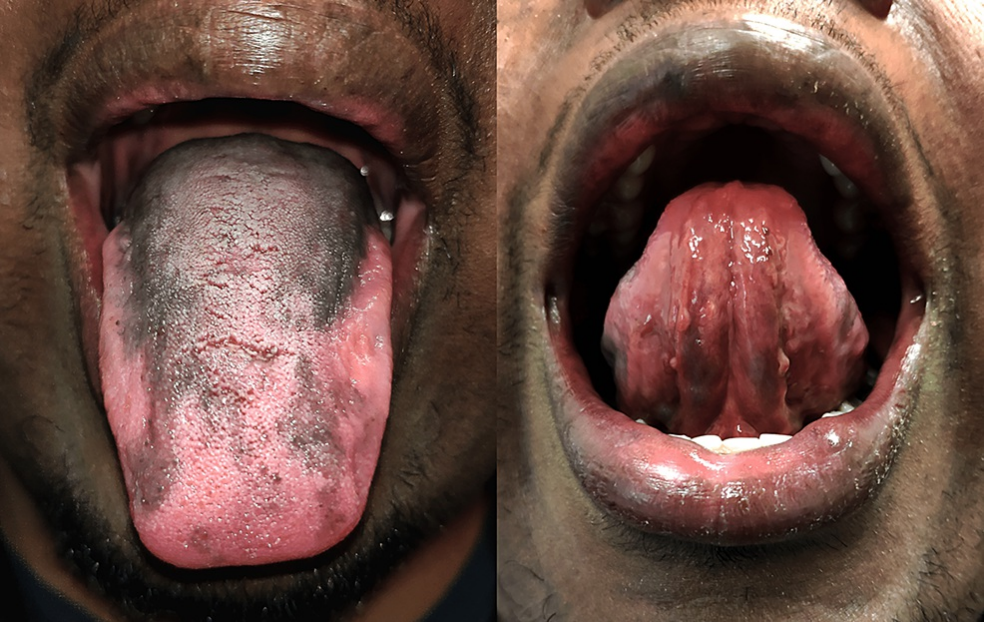
Diffuse asymmetrical brownish-black pigmentation covering most of the dorsal surface of the tongue, sparing the ventral surface.

**Figure 2:**
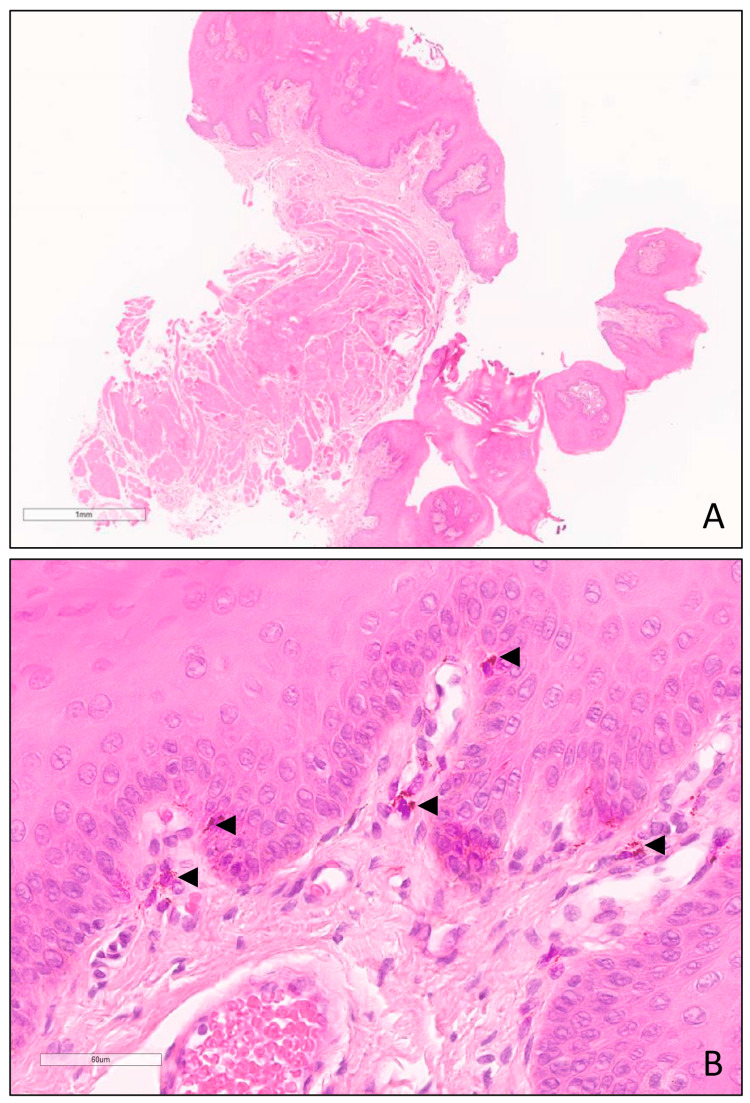
(H&E): On low-power view (A), an incisional biopsy of the tongue with globally preserved architecture can be seen. At higher power (B), melanin pigment is noted both at the basal cells of the epithelium and in the superficial connective tissue (arrowheads).

We report the case of a previously healthy 30-year-old male, Fitzpatrick skin type V, who presented with a chief complaint of extensive pigmentation of the lingual dorsum. The lesion had spontaneously appeared one year ago, with no history of oral trauma or inflammation. Color and size remained unchanged for the last six months. No other symptoms were described. The patient denied consumption of medication, alcohol, or tobacco. There were no records of melanoma or other pigmented oral lesions, intestinal polyposis, or Addison’s disease in the family. Physical examination revealed a diffuse asymmetrical brownish-black pigmentation covering most of the dorsal surface of the tongue but absent on its ventral surface (Figure 1). There were no palpable lingual masses or suspicious cervical lymph nodes. No dental restorations with amalgam were noted. No other pigmented lesions of the skin or mucosa were found on systemic examination. Because the differential diagnosis of oral brown-black pigmentation includes, among others, melanoma, an incisional biopsy of the tongue under local anesthesia was performed. Microscopic examination revealed a globally preserved tissue architecture with melanin pigment deposition at both the basal cells of the epithelium and at the lamina propria. There were no signs of malignancy (Figure 2). Clinical and histopathological findings were compatible with physiologic pigmentation of the tongue.

## Discussion

Physiologic pigmentation is a common feature that occurs mainly in non-Caucasian individuals, without gender predilection, and with a prevalence directly proportional to age [6,7]. Melanin accumulation results from increased melanocyte activity rather than melanocyte proliferation [8]. Physiologic pigmentation presents as pigmented areas with different sizes and shapes, ranging from light brown to almost black. Color may however change over time due to tobacco, hormone, or systemic medication exposure [6]. Most commonly, it is found in the attached gingiva as bilateral well-demarcated brown bands that usually spare the marginal gingiva [7]. This presentation is so typical that diagnosis is based on clinical examination alone, dismissing histological confirmation. Other oral locations afflicted with physiologic pigmentation include the buccal mucosa, lips, and palate, where pigmentation is usually more irregularly shaped with diffuse borders [9]. Physiologic pigmentation of the tongue is much less frequent and may affect the tips of the fungiform papillae on the lingual dorsum. In this report, we describe the clinical case of a 30-year-old male presenting with brownish-black pigmentation covering most of the dorsal surface of the tongue, lacking any relevant medical history or accompanying signals or symptoms. Some systemic conditions may manifest as oral pigmentation, although they do not seem to fit this particular case report. In Peutz-Jeghers syndrome, pigmented mucocutaneous macules are found on the lips, perioral areas, buccal mucosa, eyes, nostrils, fingertips, palms, soles, and perianal areas. However, they present as multiple millimetric lesions and appear in childhood, usually before the age of five, with oral pigmentation developing during the first year of life [10]. Hyperpigmentation also occurs in almost every patient afflicted with Addison’s disease. However, it is usually generalized and most prominent in sun-exposed and pressure areas. Moreover, the initial presentation of adrenal failure includes fatigue, generalized weakness, weight loss, nausea, vomiting, abdominal pain, dizziness, tachycardia, and/or postural hypotension [11]. Atypical and bizarre patterns of pigmented lesions have been associated with melanoma [12], and therefore, we decided to perform an incisional biopsy under local anesthesia. When examined microscopically (Figure 2), melanin pigment deposition was found at the basal layer of the epithelium, with incontinence to the superficial lamina propria. These features are seen in both physiologic pigmentation and melanotic macule, with the differential diagnosis being based on pigment distribution, diffuse versus focal, respectively [13]. In contrast, architectural and cytological abnormalities of the melanocytes would be expected to be found in melanoma [8]. Taking into account personal and family medical history, clinical findings, and histopathological characterization, the diagnosis of physiologic pigmentation was made. Being a benign entity, it is generally accepted that no treatment is required, unless for aesthetic purposes. Some authors have reported the successful removal of oral pigmentation, including physiologic pigmentation, using ablative lasers such as erbium-YAG [14]. Nevertheless, reports of oral melanoma arising from apparent melanotic macule and histologically benign-appearing oral melanotic lesions [15,16] suggest that follow-up of some selected patients should be considered.

## Conclusions

Pigmented lesions of the tongue can range from harmless normal variants to life-threatening conditions. A thorough investigation including patient history, clinical examination, and, if necessary, histopathological evaluation is mandatory for diagnosis.

In this case report, we described an atypical physiologic pigmentation of the tongue. Although benign in nature, its unusual presentation demanded a histological characterization. Since about one-third of oral melanoma arise from previous benign pigmented lesions, periodic evaluation of these lesions must be considered.
